# Pattern of HPV infection in basal cell carcinoma and in perilesional skin biopsies from immunocompetent patients

**DOI:** 10.1186/1743-422X-9-309

**Published:** 2012-12-17

**Authors:** Krystyna Zakrzewska, Elisa Regalbuto, Federica Pierucci, Rosaria Arvia, Sandra Mazzoli, Alessia Gori, Vincenzo de Giorgi

**Affiliations:** 1 Department of Public Health, University of Florence, Viale Morgagni 48, Florence, 50134, Italy; 2 STDs Centre, Santa Maria Annunziata Hospital, Florence, Italy; 3 Department of Dermatological Sciences, University of Florence, Florence, Italy

**Keywords:** Human papillomavirus, Beta-HPV, Non-melanoma skin cancer, Basal cell carcinoma, Squamous cell carcinoma, Perilesional skin

## Abstract

**Background:**

The association between human papillomavirus (HPV) infection and non-melanoma skin cancers (NMSCs) such as squamous cell carcinoma (SCC) and basal cell carcinoma (BCC) is not yet fully understood. We analysed the prevalence and spectrum of cutaneous beta-HPV types and mucosal/genital HPV types in paired biopsies (tumour and corresponding perilesional skin) obtained from 50 BCC immunocompetent patients. A small group of SCC patients (n=9) was also included. We also evaluated some previously postulated risk factors for HPV infection in NMSC patients.

**Results:**

All biopsies were negative for mucosal/genital HPV types. Overall, beta-HPV DNA was detected more often in SCC compared to BCC patients (78% vs 55% of total samples). The frequency of infection increased with the patient’s age [OR=4.88 (95% CI 1.29-18.39)]. There was no significant correlation between beta-HPV positivity and sex, skin type and UV exposure. The prevalence of beta-HPV species 1 types was significantly higher than those belonging to other beta-HPV species in biopsies from BCC (p=0.022) but not from SCC subjects (p=0.091). There was no significant difference in the overall prevalence of beta-HPV infection and the number of viral types between tumour lesions and perilesional skin. BCC samples were significantly more likely to be infected with beta-HPV species 1 types compared to perilesional skin (p=0.036) and showed a higher frequency of mixed infections (p=0.028).

**Conclusions:**

These findings demonstrate that beta-HPV types belonging to species 1 are the most common HPV types detected in the skin of BCC patients. Moreover beta-1-HPV types and mixed infections are significantly more frequent in tumour samples than in healthy perilesional skin. Our results suggest that beta-1-HPVs as well as co-infection with more than one viral type could be important in NMSC and in particular in BCC.

Further studies aimed to compare the biological activity of viral types in tumours and in healthy skin (viral replication and expression, interference of infection with cellular functions) are necessary to understand the role of HPV infection in skin cancer.

## Background

Human papillomaviruses (HPVs) are highly prevalent in the human population [1-3] and are classified as alpha, beta, gamma, mu and nu genera [4]. Based on the frequency of HPV-DNA detection in different kinds of lesions, HPVs can be divided into cutaneous types commonly found in benign skin warts, mucosal types detected in genital condylomas and anogenital cancers and epideromodysplasia verruciformis (EV) types, now indicated as beta-HPVs, which had initially been identified in warts and in skin carcinomas of EV patients [5].

The oncogenic role of some mucosal HPVs has been demonstrated for a number of epithelial cancers of the anogenital and upper respiratory tracts [6] while the etiological relationship between specific HPV types and non-melanoma skin cancers (NMSCs), such as squamous cell carcinoma (SCC) and basal cell carcinoma (BCC) remains unclear. A number of epidemiological studies have demonstrated the association between markers of beta-HPV infection and SCC in EV patients [7] and in organ transplant recipients [8-10]. There is accumulating evidence that beta-HPVs may play a role in the pathogenesis of NMSC also in the general, immunocompetent population [11-13]. The mechanisms by which beta-HPVs may play a role in carcinogenesis have yet to be established. One possible hypothesis is that beta-HPVs may impair host cell defences against excessive sun light exposure, thus interfering with DNA repair and apoptosis [14-16]. A lower viral load in tumour tissues than in precursor lesions such as actinic keratosis (AK) supports the “hit and run” hypothesis according to which the virus plays a role in early stages of carcinogenesis and is not necessary for the maintenance of the malignant phenotype [17].

The majority of studies on the role of HPV in NMSCs, so far, have focused on squamous cell carcinomas whereas there are a limited number of reports regarding HPV infection in basal cell carcinomas. BCC is the most common skin cancer and, in recent years, its incidence has increased worldwide [18,19]. The major risk factor for BCC is exposure to ultraviolet radiation (UVB) in individuals with susceptible skin type as well as a specific genetic background [20-22]. The role of HPV infection in the pathogenesis of BCC is poorly understood.

In the present study we analysed paired biopsies (tumour and adjacent skin) obtained from 50 BCC immunocompetent patients. A small group of SCC patients (n=9) was also included. The comparison between HPV infection pattern in tumour and in perilesional skin obtained from the same subject, and thus characterized by the same variables that influence skin cancer risk, seems to be a very good approach for assessing a possible correlation between HPV and tumours. We also evaluated some previously postulated risk factors for HPV infection in NMSC patients.

## Results

### HPV prevalence and spectrum in NMSC patients

Overall beta-HPV infection was detected in 69 out of 118 (58%) samples. The infection was found to be more prevalent in samples from SCC patients [14 out of 18 (78%)] in comparison to BCC patients [55 out of 100 (55%)], however this difference did not reach statistical significance (p=0.058) (Table 1). The number of positive samples detected using the RHA Kit Skin (beta) HPV corresponded to that obtained by the nested FAP-PCR. All specimens that tested negative for HPV DNA were positive for human beta-globin DNA, indicating that these samples contained DNA of sufficient quantity and quality for PCR amplification. All biopsies resulted negative for mucosal/genital HPVs.

**Table 1:** Prevalence of human beta-papillomavirus (beta-HPV) in NMSC and in perilesional skin biopsies in immunocompetent patients

**Diagnosis**	**Tumour biopsy**	**Perilesional skin**	**Total**
	**No. of positive/total (%)**	**No. of positive/total (%)**	**No. of positive/total (%)**
BCC	31/50 (62)	24/50 (48)	55/100 (55)
SCC	8/9 (89)	6/9 (67)	14/18 (78)
Total	39/59 (66)	30/59 (51)	69/118 (58)

*NMSC*: non-melanoma skin cancer; *BCC*: basal cell carcinoma; *SCC*: squamous cell carcinoma.

Forty-four out of 59 (75%) patients harboured beta-HPV DNA in tumour and/or in perilesional skin. Thirty-five out of 50 (70%) BCC patients and 9 out of 9 (100%) SCC patients resulted positive at tumour and/or at perilesional skin. There was no statistically significant difference with regard to HPV prevalence between SCC and BCC patients (p=0.056).

In the analysed samples we detected beta-HPV types belonging to species 1 (HPV5, 8, 12, 14, 19, 20, 21, 24, 25, 36, 47, 93, 105), species 2 (HPV9, 15, 17, 23, 37, 38, 80, 100), species 3 (HPV49), species 4 (HPV92), and some previously described putative unclassified types (FA7, FA14, FA108, FA114.2, FA118, FA149, FAIMVS6.4, FAIMVS7). The most prevalent types (HPV5, 8, 24 and HPV93), all belonging to the beta-HPV species 1, were present in 18%, 10%, 15%, and 10% of total samples respectively. HPV types 5, 8 and 93 were found in both tested groups whereas HPV24 was present in samples from BCC patients only.

Viral types within beta-HPV species 1 were significantly more common, found in 60 out of 118 (51%) samples, in comparison to those belonging to other beta-HPV species which were detected in 40 out of 118 (34%) biopsies (p=0.006) (Table 2). Among biopsies obtained from BCC patients beta-1-HPV types were significantly more frequent [48 out of 100 (48%) samples] than other beta-HPV types [33 out of 100 (33%) samples] (p=0.022). Among biopsies obtained from SCC patients beta-1-HPV types were more frequent [12 out of 18 (67%) samples] than other beta-HPV types [7 out of 18 (39%) samples] but this difference did not reach statistical significance (p=0.091).

**Table 2:** Prevalence of beta-HPV species and frequency of multiple infections in tumour and in perilesional skin samples from NMSC immunocompetent patients

**Diagnosis**	**Tumour biopsy**	**Perilesional biopsy**	**Total**
	**No. of positive/total (%)**	**No. of positive/total (%)**	**No. of positive/total (%)**
		Beta1-HPV	
BCC	29/50 (56)	19/50 (38)	48/100 (48)
SCC	7/9 (76)	5/9 (56)	12/18 (67)
Total	36/59 (61)	24/59 (41)	60/118 (51)
		Other than beta1-HPV	
BCC	18/50 (36)	15/50 (30)	33/100 (33)
SCC	5/9 (56)	2/9 (22)	7/18 (39)
Total	23/59 (39)	17/59 (29)	40/118 (34)
		Multiple infection	
BCC	22/50 (44)	12/50 (24)	34/100 (34)
SCC	6/9 (67)	4/9 (22)	10/18 (56)
Total	28/59 (47)	16/59 (27)	44/118 (37)

*NMSC*: non-melanoma skin cancer; *BCC*: basal cell carcinoma; *SCC*: squamous cell carcinoma.

Forty-four out of 118 (37%) samples harboured more than one genotype. There was no statistically significant difference with regard to the prevalence of mixed infections between samples from BCC and SCC patients [34 out of 100 (34%) vs 10 out of 18 (56%); p=0.072] (Table 2).

The frequency of beta-HPV infection in the studied population increased with the patient’s age when adjusted for sex, skin type and UV exposure (OR=4.88 [95% CI, 1.29-18.39]) (Table 3). When analysis was performed separately for BCC patients the adjusted OR was 4.6 [95% CI, 1.1-18.3]. There was no significant correlation between beta-HPV positivity and sex, skin type and UV exposure.

**Table 3:** Risk factors for cutaneous human papillomavirus infection in NMSC subjects

**HPV status**				
**No. of positive /total subjects**				
**Category**	**BCC Positive (no. = 35)**	**SCC Positive (no. = 9)**	**Total Positive (no. = 44)**	**Adjusted OR (95% CI)**^**a**^
Sex				
Female	16/22	3/3	19/25	
Male	19/28	6/6	25/34	1.11 (0.30-4.05)
Age				
48-70	7/15	1/1	8/16	
71-99	28/35	8/8	36/43	4.88 (1.29-18.39)
Skin type				
3	23/34	4/4	27/38	
2	12/16	5/5	17/21	0.82 (0.20-3.37)
UV exposure				
Low	18/27	7/7	25/34	
Moderate/high	17/23	2/2	19/25	0.59 (0.16-2.10)

*NMSC*: non-melanoma skin cancer; *BCC*: basal cell carcinoma; *SCC*: squamous cell carcinoma.

^a^ OR values were calculated for the total (no.= 44) positive subjects and adjusted for all variables in the table.

### Comparison between NMSC and perilesional skin biopsies

Beta-HPV DNA was detected more often in tumours [39 out of 59 (66%)] than in perilesional skin [30 out of 59 (51%)], however this difference did not reach statistical significance (p=0.067). Thirty-one out of 50 (62%) BCC samples and 24 out of 50 (48%) corresponding perilesional skin biopsies tested beta-HPV positive and among samples obtained from SCC patients 8 out of 9 (89%) tumours and 6 out of 9 (67%) paired perilesional skin resulted beta-HPV positive (Table 1). No statistically significant difference in HPV prevalence was observed between tumours and perilesional skin from BCC (p=0.114) and from SCC patients (p=0.288).

Among 59 tumor/perilesional skin biopsy pairs 15 (25%) were HPV negative, 19 (32%) patients were positive only at tumour or at perilesional skin while 25 (42%) had beta-HPV DNA in both biopsies. In 2 patients the viral types in tumours were different from those detected in perilesional skin, 3 patients had identical HPV types in both biopsies and in 20 patients one or more identical HPV types were detected in tumours and in corresponding healthy skin.

**Figure 1:**
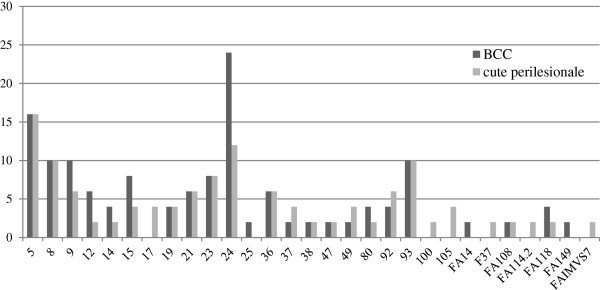
**Comparison of the spectrum and prevalence of beta-HPV types in BCC and in perilesional skin biopsies.** The percentage of positive samples harbouring each beta-HPV type/putative type is illustrated. Many biopsies contained more than one HPV type.

Among the most prevalent types, HPV5, 8 and 93 were uniformly distributed between tumours and perilesional skin samples while HPV24 was twice as frequent in BCC than in corresponding perilesional skin. No particular beta-HPV types were significantly associated tumours or with healthy tissue (Figure 1).

Thirty-six out of 59 (61%) tumours tested positive for beta-1-HPV types compared with 23 out of 59 (39%) tumours positive for other beta-HPV types. This difference was statistically significant (p=0.013) (Table 2). Viral types belonging to species 1 were significantly more frequent in tumours than in perilesional skin [36 out of 59 (61%) vs 24 out of 59 (41%); p=0.021]. Moreover the prevalence of beta-1-HPV in samples from BCC patients was significantly higher than the prevalence of other beta-HPV types [29 out of 50 (56%) vs 18 out of 50 (36%); p=0.022] and was also significantly higher in tumours than in perilesional skin [29 out of 50 (56%) vs 19 out of 50 (38%); p=0.036].

Infections with multiple HPV types was found to be significantly more common in tumours [28 out of 59 (47%)] than in perilesional skin [16 out of 59 (27%)] (p=0.018). Mixed infections were detected in 22 out of 50 (44%) BCC samples compared with 12 out of 50 (24%) of corresponding perilesional skin biopsies. This difference was statistically significant (p=0.028) (Table 2).

Viral load determination by real-time PCR for HPV5 and HPV24 revealed low HPV DNA levels, frequently below the detection limit of quantitative PCR, both in tumour and in perilesional skin biopsies. In samples with quantifiable DNA levels the viral loads ranged from 1 viral copy per 12,000 cell equivalents to 1 copy per 0.01 cell equivalents (100 HPV DNA copies per 1 cell equivalent). The comparison of HPV5 DNA level in tumour and perilesional skin obtained from 3 patients revealed a 20–50 times higher viral load in tumours than in perilesional skin. On the other hand, HPV24 DNA level was 20–60 times higher in perilesional skin than in tumour samples obtained from 4 patients (Table 4).

**Table 4:** Viral load determination for HPV5 and HPV24 in paired samples (tumour and corresponding perilesional skin)

**Patient**	**1 HPV5 copy/no. of cell**		**Patient**	**1 HPV24 copy/no. of cell**	
	**equivalents**^**1**^			**equivalents**^**1**^	
	BCC	Perilesional skin		BCC	Perilesional skin
1	61	1200	4	75	2.3
2	1	50	5	6410	107
3	132	3566	6	641	32
-	-	-	7	932	22

^1^ The viral loads were corrected for the input amount cells as described in “Material and methods”.

## Discussion

The overall beta-HPV positivity in the tested population was high since 70% of BCC patients had viral DNA detected in the tumour and/or perilesional skin. A higher prevalence of infection (100%) was observed in the SCC group, but this did not reach statistical significance. All samples were negative for the high risk and low risk HPV DNA screening and genotyping test able to detect 28 main mucosal/genital HPV types. This could underscore the propensity of certain HPV types to infect and replicate preferentially in either mucosal or cutaneous epithelium and is in line with some previous studies, where the mucosal types were not found at all or only rarely either in tumour or normal skin samples [8,9,23,24]. On the other hand, the findings of Iftner and colleagues [25] have suggested that persistent infections of the skin with some high risk genital HPV types such as HPV16, 31, 35 and 59 may be a risk factor for NMSC in the immunocompetent population. These discrepancies between results obtained in different investigations could reflect differences in sensitivity and/or specificity of detection methods used by various authors as well as differing characteristics of patients included in the studies. Since the commercial test used in our study is highly sensitive and specific we can conclude that genital HPV types, if present, are extremely rare in the skin disorders, which were the subject of the present study.

We observed that increasing age was associated with a higher prevalence of beta-HPV infection in our studied population. This observation is in line with some data reported in the literature [3] and may be the consequence of the natural deterioration of the immune system which could facilitate both viral infection and reactivation in elderly individuals. There was no significant correlation between beta-HPV positivity and sex, skin type and UV exposure.

We found no significant differences in HPV prevalence between tumours and perilesional skin samples. However, viral DNA was detected more often in both BCC and SCC than in corresponding perilesional skin biopsies. These findings are in line with some previous studies in which the prevalence of HPV infection in both kinds of tumours was slightly higher than in adjacent perilesional [24,26]. The detection rate of HPV DNA in tumour samples analysed in our study falls within the value range reported in other investigations [13,23]. It is also higher than the frequency of beta-HPV detection in normal skin from healthy subjects [25,27].

We found that beta-1-HPV types were significantly more common than types belonging to other beta-HPV species (p=0.006) in our studied population. This was also observed in the case of samples from BCC (p=0.022) but not from SCC patients. The most prevalent types (HPV5, 8, 24 and 93) identified in samples from BCC patients accounted for 41% (54 out of 133) of total infections. HPV24 was never detected in SCC samples and the most prevalent types (HPV5, 8 and 93) represented 26% (9 out of 34) total infections. Some literature data indicate that beta-HPV species 2 is more likely to be identified in SCC than in perilesional healthy skin [23,26]. In another study beta-HPV species 1 types were detected more often in SCC than in BCC biopsies [13]. In the present investigation we mainly analysed paired tumour and perilesional skin biopsies from BCC patients. Only a limited number of samples from SCC individuals was included. We found that the genotypes belonging to species 1 were statistically more common in tumours than in perilesional skin from the overall studied population (p=0.021) and this was also the case with BCC patients alone (p=0.036).

The prevalence of mixed infections was found to be high since 44 out of 69 (64%) positive samples harboured more than one beta-HPV type. Multiple infections were more common in samples from SCC patients (56%) compared to samples from BCC patients (34%) although this difference was not statistically significant. Mixed infections were significantly higher in tumours than in perilesional skin (p=0.018) of the overall studied population and of BCC patients alone (p=0.028).

Twenty-five out of 44 beta-HPV positive patients (57%) harboured HPV infection in both biopsies whereas 19 (43%) patients had only positive tumour or perilesional samples.

There was no significant difference in the number of viral types between tumours and adjacent skin. We observed a high degree of overlap between the beta-HPV types in paired biopsies as the viral types found in tumours were completely different from those present in perilesional skin from the same patients in only in 2 out of 25 cases. Our results differ somewhat from the findings of a study of HPV prevalence and spectrum in SCC patients carried out by Plasmeijer and collaborators [28]. In this study quite a similar median number of beta-HPVs was found in perilesional skin and SCC tissue, but a different spectrum of viral types was seen. The authors hypothesized that the types present in the perilesional skin could partially represent commensal types, whereas those in the tumour could be associated with SCC formation. In agreement with previous studies [24,28] we found that no single type prevailed in NMSC and/or in perilesional skin in immunocompetent individuals. Moreover, HPV24 was twice as frequent in BCC than in perilesional skin.

A number of studies report the prevalence and distribution of HPV types in NMSCs, in benign lesions and in perilesional skin but there are limited data regarding the rate of replication and expression of different beta-HPVs in different kinds of lesions*.* It cannot be excluded that different beta-HPVs may exhibit different rates of replication and/or different expression patterns in different types of lesions such as tumour and normal perilesional skin. A comparison of HPV DNA loads in NMSC and in corresponding adjacent skin described in previous studies [24,29] did not reveal the tendency to differ. Interestingly a comparison of viral load in a limited number of paired biopsies both positive for HPV5 or for HPV24, carried out in our study, might suggest a varying replicative activity of two viral types in two different kinds of samples.

Further studies need to be performed to investigate the biological activity of viral types detected in paired biopsies. It can be useful to determinate viral load, viral gene expression and the interference of HPV infection with cellular gene expression in tumours and in healthy skin to understand the role of HPV infection in skin cancers. A recent study has demonstrated that the up-regulation of p16^INK4a^ and AKT/P13K pathway in BCC is often associated with the presence of beta-HPV species 2 suggesting, that in a subtype of BCC these viruses may exert a role in the carcinogenesis or in other, still undefined, biological property of these tumours [30].

## Conclusions

In summary, the overall prevalence of beta-HPV was higher in SCC than in BCC patients. No mucosal/genital HPV types were detected. Beta-HPV types belonging to species 1 represented the most common infection detectable in NMSC, and in particular, in the BCC population. The most common types detected in samples from the BCC patients (HPV5, 8, 24 and 93) accounted for 41% of total infections. The tumour lesions did not seem to differ significantly from perilesional skin with regard to the overall prevalence of beta-HPV infection and the number of viral types detected in paired biopsies. However beta-HPV types from species 1 were significantly more frequent in BCC samples which were also characterized by a higher frequency of multiple infections. Our results suggest that beta-1-HPVs as well as co-infection with more than one viral type could be important in NMSC and in particular in BCC.

## Material and methods

### Patients

All procedures followed were approved by appropriate Ethics Committee related to our institutions (Azienda Sanitaria di Firenze, Università di Firenze, Firenze, Italy). Research was carried out in accordance with Helsinki Declaration. All patients were recruited after written, informed, consent. Fifty-nine patients, 25 female and 34 male, referred to the Dermatology Clinic at the University of Florence for the removal of suspected NMSCs were studied. All patients were immunocompetent (mean age 75.7 years, range 48–99). None of the patients was receiving immunosuppressive or immunostimulating drug therapy.

### Samples

Punch biopsies were collected from both the tumour and perilesional skin (roughly 1 cm away from the lesional border) from each patient. The samples were immersed in RNA*later* RNA Stabilization Reagent (Qiagen) and conserved at −20°C. The remaining tumour was excised and sent for histopathological diagnosis.

Overall, 50 BCC patients and 9 SCC patients were included in this study. The samples were classified on the basis of skin type (II-III, according to the Fitzparick classification [31]). The level of sun exposure at the site of biopsy was classified, by a single dermatologist, into three categories based on anatomical site: extensive (i.e., head and neck), moderate (i.e., trunk and extremities), or low (i.e., buttocks and genital area).

### Nucleic acids extraction

The DNA was extracted from each sample using All Prep DNA/RNA Mini Kit (Qiagen) and stored at −70°C. The quality of isolated DNA was checked by PCR amplification of the human beta-globin gene [32].

### Beta-HPV DNA detection and typing

To maximize the reliability and sensitivity of analysis, the samples were tested by two different methods. First the two-step version of the nested FAP-PCR with degenerated primers was performed. This PCR yielded a 235-base pair fragment corresponding to part of an L1 gene and is able to amplify more than 20 known HPV types, mainly beta-HPVs [33]. The PCR products were purified using DNA gel extraction kit (Millipore) and cloned using a pGEM-T Easy Vector System (Promega). At least 2 clones per sample were purified using QIAprep Spin Miniprep Kit (Qiagen) and sequenced according to the manual of the Big Dye Terminator Cycle Sequencing kit using the ABI310 sequencer (Applied Biosystems). The sequencing reaction was performed using primers flanking the plasmid cloning region: forward primer T7 5’-TAA TAC GAC TCA CTA TAG GG and reverse primer Sp6 5’-TAT TTA GGT GAC ACT ATA G. For genotyping the DNA sequences were compared with those already available in GenBank database, by using the BLAST server (http://blas.ncbi.nlm.nih.gov//Blast.cgi).

Subsequently HPV detection and genotyping were carried out using the RHA Kit Skin (beta) HPV (Diassay, Rijswijk, the Netherlands). This method was designed for the amplification and typing of 25 beta-HPV types. Briefly, a broad spectrum PCR using biotinylated consensus primers was used to amplify a 117-base pair region from the E1 gene. The biotinylated amplicons were subsequently hybridized with specific oligonucleotide probes immobilized as parallel lines on membrane strips. The bands were then analyzed against a reference sheet.

The necessary precautions to avoid contamination between samples were observed during all steps of sample handling and, in each PCR run, water samples and known HPV negative specimens were included as negative controls.

### Mucosal/genital HPV detection

All samples were analysed by INNO-LiPA HPV Genotyping Extra (Innogenetics, Italy) according to the manufacturer’s instructions. INNO-LiPA HPV Genotyping Extra is a line probe assay, based on the reverse hybridization principle, designed for the identification of 28 different genotypes of the human papillomavirus by detection of specific sequences in the L1 region of the HPV genome. The assay covers all currently known high-risk HPV genotypes and probable high-risk HPV genotypes (16, 18, 26, 31, 33, 35, 39, 45, 51, 52, 53, 56, 58, 59, 66, 68, 73, 82) as well as a number of low-risk HPV genotypes (6, 11, 40, 43, 44, 54, 70) and some additional types (69, 71, 74).

### Statistical analysis

Stata software was used to analyse the data by logistic regression analysis. Fisher’s exact test was used for analysis of the differences in distribution of HPV prevalence among groups. A P<0.05 was considered to be statistically significant.

### HPV DNA quantification

Type-specific real time PCR protocols described previously [34] were used for HPV5 and 24 DNA quantification. To check for PCR efficiency and DNA integrity and to determine the number of input cell equivalents, the beta-globin gene was quantified. The HPV DNA loads were defined as 1 HPV DNA copy per number of cell equivalents. Two beta-globin gene copies were taken as a cell equivalent. Some negative controls (total cellular DNA) were included in each run.

## Abbreviations

BCC: Basal cell carcinoma; HPV: Human papillomavirus; NMSC: Non-melanoma skin cancer; SCC: Squamous cell carcinoma; AK: Actinic keratosis.

## Competing interests

The authors declare that they have no competing interests.

## Authors’ contribution

KZ conceived and planned the study and wrote the manuscript, ER and FP carried out the sample extraction, amplification and typing, RA carried out the quantitative assay, SM took mucosal HPV detection and helped to draft the manuscript, AG and VDG provided clinical samples and helped to draft the manuscript. All authors have read and approved the final version of the manuscript.
